# A novel QTL *GSE3.1* regulates grain size and weight in rice

**DOI:** 10.3389/fpls.2026.1784176

**Published:** 2026-03-11

**Authors:** Kaiying Wang, Jiahao Dong, Chenjie Wang, Chen Zhou, Zhao Li, Wenjie Jiao, Limin Zhang, Minying Dou, Nannan Han, Baolan Zhang, Ran Xu, Yunhai Li, Zupei Liu, Luojiang Huang

**Affiliations:** 1 School of Breeding and Multiplication (Sanya Institute of Breeding and Multiplication), Hainan University, Sanya, China; 2 State Key Laboratory of Seed Innovation, Institute of Genetics and Developmental Biology, Chinese Academy of Sciences, Beijing, China; 3 Key Laboratory of Forage and Endemic Crop Biotechnology, Ministry of Education, School of Life Sciences, Inner Mongolia University, Hohhot, China; 4 College of Agriculture and Biotechnology, Zhejiang University, Hangzhou, China

**Keywords:** grain size, grain weight, GSE3.1, QTL, rice

## Abstract

Grain size is a crucial agronomic trait that directly determines grain yield. To elucidate molecular mechanisms of grain size control will help breeders develop high-yield varieties. In this study, we report a novel quantitative trait locus (QTL) *GRAIN SIZE AND WEIGHT ON CHROMOSOME 3* (*GSE3.1*) for grain size and weight in rice, which encodes a TONNEAU1-Recruiting Motif family (TRM) protein and positively regulates grain size by promoting cell division. In addition, the *GSE3.1* allele from Daligeng (DLG) significantly increases grain size and weight, and further improves grain yield in field plot trials. Thus, our findings demonstrate the molecular mechanism of *GSE3.1* in grain size and weight control, suggesting this gene could be a promising target for breeding high-yield varieties in rice.

## 1 Introduction

Rice is one of the most important cereal crops worldwide, and feeds more than half of the world’s population (Khush, 2001). Improving rice yield is of vital importance for ensuring global food security. As one of three key determinants of rice yield, grain weight is positively related to grain size (Zuo and Li, 2014). During the past decades, constant efforts have been made by researchers to clone and characterize grain size-related genes, most of which are involved in transcriptional regulation, G protein signaling, plant hormone signaling, the MAPK signaling pathway, and ubiquitin proteasome pathway (Li et al., 2019). Nevertheless, our understanding of the genetic and molecular mechanisms in grain size control is still limited but crucial for improving grain yield.

As plant cytoskeletal proteins, microtubules are indispensable regulators of cell division since diverse microtubule arrangements are associated with distinct stages of the cell cycle (Ehrhardt and Shaw, 2006). During the process of cell division, formation of preprophase band (PPB) of microtubules is the premitotic cytological landmark of the final division plane, and PPB disassembles in late prophase, progressively replaced by a mitotic spindle during metaphase and anaphase (Ambrose and Cyr, 2008). Therefore, precise assembly and disassembly of PPB can ensure the accurate segregation of genetic material and the formation of daughter cells. The PPB assembly is controlled by the TON1/TRM/PP2A (TTP) complex (Schaefer et al., 2017). In Arabidopsis, TRM proteins interact with and target TONNEAU1 (TON1) and type 2A protein phosphatase subunits (PP2A) to cortical microtubules to form the TTP complex, revealing their crucial roles in plant cell division (Drevensek et al., 2012; Schaefer et al., 2017; Spinner et al., 2013). Mutants of TRM genes impair the formation of PPB, which causes inaccuracy in cell division orientation (Schaefer et al., 2017). Up to date, only a few TRM proteins have been reported to participate in plant organ developmental processes (Lee et al., 2006; Wang et al., 2023, 2015, 2015; Wu et al., 2018; Zhou et al., 2015). Arabidopsis *TRM1*/*LNG2* and *TRM2*/*LNG1* regulate organ elongation by positively promoting longitudinal polar cell elongation (Lee et al., 2006). Rice *GRAIN LENGTH ON CHROMOSOME 7* (*GL7*)/*GRAIN WIDTH 7* (*GW7*)/*SLENDER GRAIN ON CHROMOSOME 7* (*SLG7*) encodes a TRM protein, which interacts with OsTON1b and OsTON2, the other two components in TTP complex, and gain-of-function *GL7*/*GW7*/*SLG7* results in slender grains (Wang et al., 2015, 2015; Wu et al., 2023; Zhou et al., 2015). Therefore, more studies are needed to elucidate molecular mechanisms of TRM proteins in organ size control, which are beneficial for increasing seed/grain weight and yield.

In this study, we performed fine mapping of a novel QTL controlling grain size and weight in rice, and identified the causal gene *GSE3.1*, which has been previously reported to regulate grain size (Niu et al., 2024; Wu et al., 2023). *GSE3.1* encodes a TRM protein, which simultaneously promotes grain length and grain width by positively regulating cell proliferation in both longitudinal and transverse directions in the spikelet hulls. We also demonstrate that the *GSE3.1*^DLG^ allele, with higher expression of *GSE3.1*, leads to increased grain size and weight, and significantly increased grain yield in the field plot trials, which suggests its application potential for improving rice grain yield.

## 2 Results

### 2.1 Map-based cloning of *GSE3.1*

**Figure 1:**
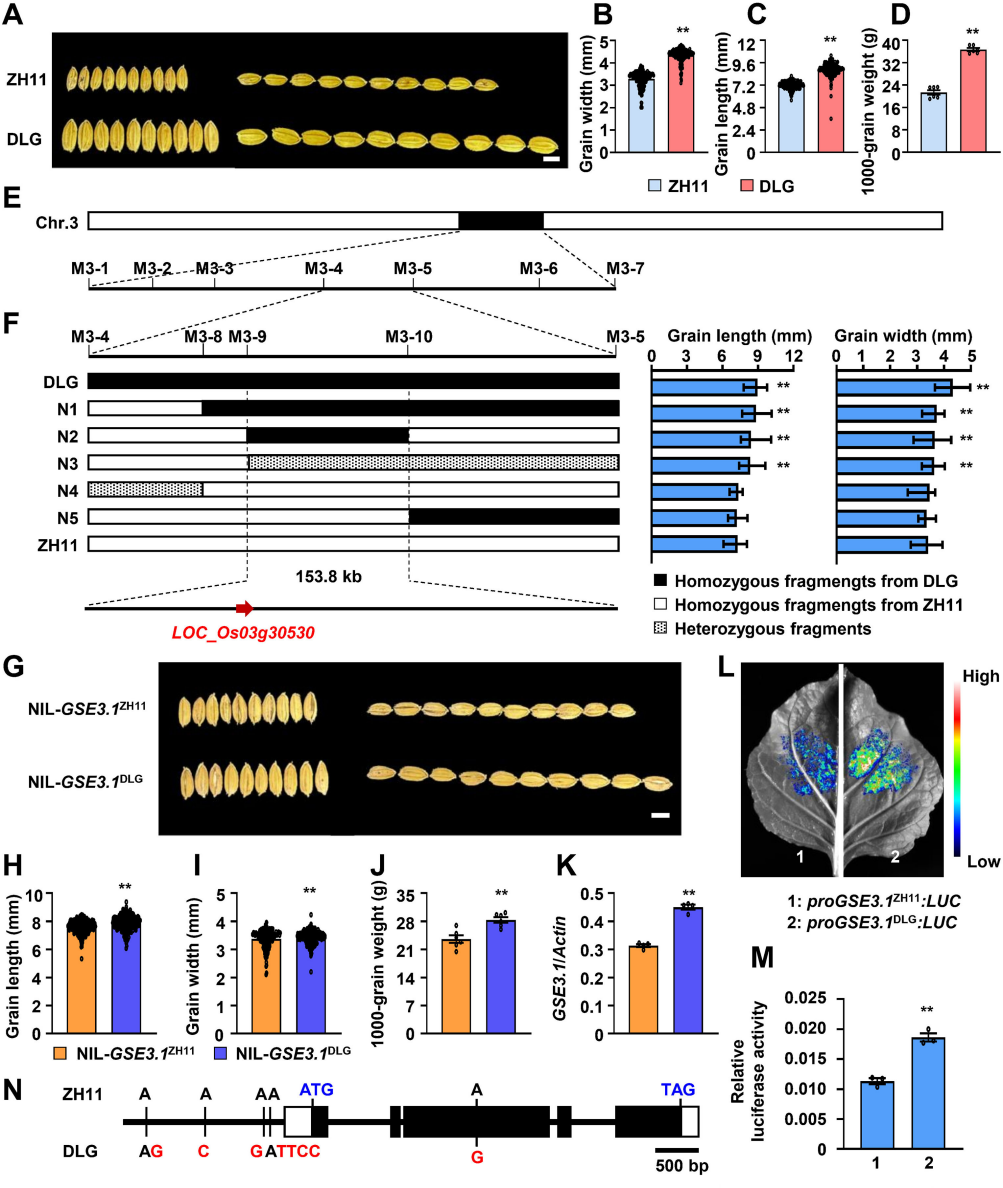
Map-based cloning *GSE3.1*. **(A)** mature rice grains of ZH11 and DLG. **(B)** grain width of ZH11 and DLG (n ≥ 170). **(C)** grain length of ZH11 and DLG (n ≥ 170). **(D)** 1,000-grain weight of ZH11 and DLG (n = 6). **(E, F)** fine mapping of *GSE3.1*. Left: high-resolution mapping. Right: grain length and grain width analyses of recombinants. **(G)** mature rice grains of NIL-*GSE3.1*^ZH11^ and NIL-*GSE3.1*^DLG^. **(H)** grain length of NIL-*GSE3.1*^ZH11^ and NIL-*GSE3.1*^DLG^. (N ≥ 210). **(I)** grain width of NIL-*GSE3.1*^ZH11^ and NIL-*GSE3.1*^DLG^ (n ≥ 210). **(J)** 1000-grain weight of NIL-*GSE3.1*^ZH11^ and NIL-*GSE3.1*^DLG^ (n = 6). **(K)** relative expression levels of *GSE3.1* in young panicles of NIL-*GSE3.1*^ZH11^ and NIL-*GSE3.1*^DLG^ (n = 4). **(L, M)** activity analyses of *GSE3.1* promoters from ZH11 and DLG by using the dual-luciferase reporter system. The 2.3 kb *GSE3.1* promoter sequences from ZH11 and DLG were cloned upstream of the firefly luciferase (LUC) gene to generate the *proGSE3.1*^ZH11^*:LUC* and *proGSE3.1*^DLG:^*LUC* constructs, which were transiently transformed into *N. Benthamiana* leaves. After 48 h, the image was captured, indicating *proGSE3.1*^DLG:^*LUC* has a higher transcription activity **(L)**. In addition, A renilla luciferase (REN) gene driven by the 35S promoter served as an internal control, and the ratio of LUC to REN luminescence indicates the relative luciferase activity. The diagram shows *proGSE3.1*^DLG:^ LUC has higher relative luciferase activity than *proGSE3.1*^ZH11^:LUC **(M)**. **(N)** DLG has four variations in the *GSE3.1* promoter, and one variation in the coding sequence compared to ZH11. The variations are shown in red and start and stop codons are shown in blue. Values **(B–D, H–K, M)** are given as mean ± SE. **P < 0.01 compared with ZH11, NIL-*GSE3.1*^ZH11^, or *proGSE3.1*^DLG:^ LUC by Student’s *t*-test. Bars, 5 mm **(A, G)**.

To explore QTLs for rice grain size, we selected the japonica variety DLG as a donor and crossed it with the japonica variety Zhonghua11 (ZH11). Compared to ZH11, DLG had significantly increased grain length and grain width, thus resulting in large and heavy grains (**Figures 1A–D**). We sequenced the whole genomes of ZH11 and DLG, and designed specific markers for mapping (**Supplementary Table 3**). The F_2_ population was used to map a major QTL *GSE3.1* responsible for grain size and weight on chromosome 3. The *GSE3.1* locus was further fine-mapped to the 153.8 kb genomic region by using the 2168 BC_3_F_3_ individuals (**Figures 1E, F**).

To identify the candidate gene responsible for the grain size variation between ZH11 and DLG, we carefully compared the genomic sequences of ZH11 and DLG within the 153.8 kb interval and found that three of the total 26 genes contain sequence variations in the promoter, CDS, exon-intron boundary, 5’ UTR and 3’ UTR regions (**Supplementary Tables 1, 2**). Compared with ZH11, DLG contains four variations in promoter and one variation in CDS in the *LOC_Os03g30530* region and has one variation in 3’ UTRs of *LOC_Os03g30550* and *LOC_Os03g30580* (**Figure 1N** and **Supplementary Table 2**). Next, near-isogenic lines (NILs) were obtained in the BC_4_F_3_ population and subjected to further expression analyses. Compared to NIL-*GSE3.1*^ZH11^, NIL-*GSE3.1*^DLG^ showed increased grain length, width and weight (**Figures 1G–J**). Strikingly, expression of *LOC_Os03g30530* in NIL-*GSE3.1*^DLG^ was significantly higher than that in NIL-*GSE3.1*^ZH11^, while no obvious expression differences of *LOC_Os03g30550* and *LOC_Os03g30580* were detected (**Figure 1K**; **Supplementary Figure 1**). Since there were four variations in the promoter region of *LOC_Os03g30530* between ZH11 and DLG, we investigated the activities of the *LOC_Os03g30530* promoters from ZH11 and DLG (*proGSE3.1*^ZH11^ and *proGSE3.1*^DLG^) in *N. benthamiana* leaves by using the luciferase reporter system. As shown in **Figures 1L, M**, the *proGSE3.1*^DLG:^*LUC* had stronger luciferase activity than *proGSE3.1*^ZH11^*:LUC*, suggesting the *LOC_Os03g30530* promoter from DLG resulted in higher expression of *GSE3.1*. Therefore, *LOC_Os03g30530* is the candidate gene for *GSE3.1*.

### 2.2 *GSE3.1* positively regulates grain size and weight

**Figure 2:**
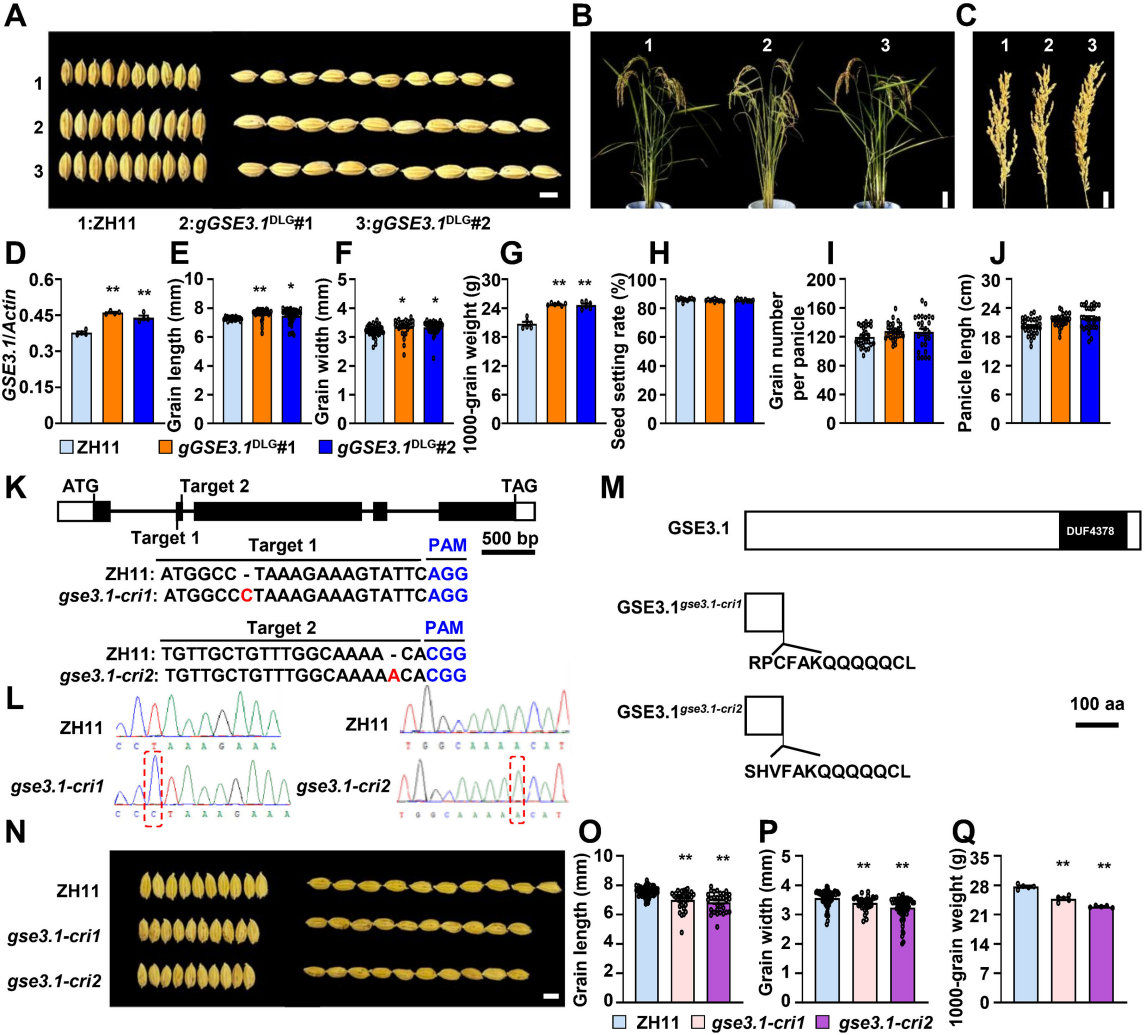
*GSE3.1* positively regulates grain size and grain weight. **(A)** mature rice grains of ZH11, *gGSE3.1*^DLG^#1 and *gGSE3.1*^DLG^#2. **(B)** plant morphology of ZH11, *gGSE3.1*^DLG^#1 and *gGSE3.1*^DLG^#2. **(C)** panicle morphology of ZH11, *gGSE3.1*^DLG^#1 and *gGSE3.1*^DLG^#2. **(D)** relative expression levels of *GSE3.1* in young panicles of ZH11, *gGSE3.1*^DLG^#1 and *gGSE3.1*^DLG^#2. (N = 4). **(E, F)** grain length **(E)** and grain width **(F)** of ZH11, *gGSE3.1*^DLG^#1 and *gGSE3.1*^DLG^#2. (N ≥ 40). **(G)** 1000-grain weight of ZH11, *gGSE3.1*^DLG^#1 and *gGSE3.1*^DLG^#2. (N = 6). **(H)** seed setting rate of ZH11, *gGSE3.1*^DLG^#1 and *gGSE3.1*^DLG^#2. (N ≥ 10). **(I)** grain number per panicle of ZH11, *gGSE3.1*^DLG^#1 and *gGSE3.1*^DLG^#2. (N ≥ 25). **(J)** panicle length of ZH11, *gGSE3.1*^DLG^#1 and *gGSE3.1*^DLG^#2. **(K)** schematic of the *GSE3.1* gene structure. The start codon (ATG) and stop codon (TAG) are indicated. Black boxes represent exons, white boxes indicate the 5´- and 3´- untranslated regions, and the lines between boxes denote introns. The two target sites (Target 1 and Target 2) are shown, and the PAM sequence are shown in blue. The mutations in *gse3.1-cri1* and *gse3.1-cri2* are shown in red. **(L)** sequencing diagrams showing the mutations in gse3.1-cri1 and gse3.1-cri2. The red dotted boxes indicate the insertions. (M) protein structure of GSE3.1. Mutated GSE3.1 proteins of gse3.1-cri1 and gse3.1-cri2 are shown. **(N)** mature rice grains of ZH11, *gse3.1-cri1* and *gse3.1-cri2*. **(O, P)** grain length **(O)** and grain width **(P)** of ZH11, *gse3.1-cri1* and *gse3.1-cri2* (n ≥ 30). **(Q)**, 1000-grain weight of ZH11, *gse3.1-cri1*, and *gse3.1-cri2* (n = 6). Values **(D–J, O–Q)** are given as mean ± SE. **P < 0.01 and *P < 0.05 compared with ZH11 by Student’s *t*-test. Bars, 5 mm **(A, N)**, 15 cm **(B)**, 3 cm **(C)**, 500 bp **(K)**, 100 aa **(L)**.

To further validate whether *LOC_Os03g30530* is the *GSE3.1* QTL, we carried out a genomic transformation test. A genomic fragment (*gGSE3.1*^DLG^) from the DLG variety was transformed into ZH11. The *gGSE3.1*^DLG^ transgenic plants exhibited significantly increased grain length, grain width and 1000-grain weight (**Figures 2A, E–G**). Next, *LOC_Os03g30530* was knocked out by using the CRISPR/Cas9 system and two knockout lines, *gse3.1-cri1* and *gse3.1-cri2*, were obtained. *gse3.1-cri1* and *gse3.1-cri2* had one base pair insertion in the first and second exon of the *GSE3.1* gene (**Figures 2K, L**), respectively, which resulted in a premature stop codon and truncated GSE3.1 proteins (**Figure 2M**). Compared to ZH11, both *gse3.1-cri1* and *gse3.1-cri2* showed decreased grain length, grain width and 1000-grain weight (**Figures 2N–Q**). Taken together, *LOC_Os03g30530* is the causal gene of *GSE3.1*, and *GSE3.1* positively regulates grain size and weight.

### 2.3 *GSE3.1* promotes cell division to regulate grain size

**Figure 3:**
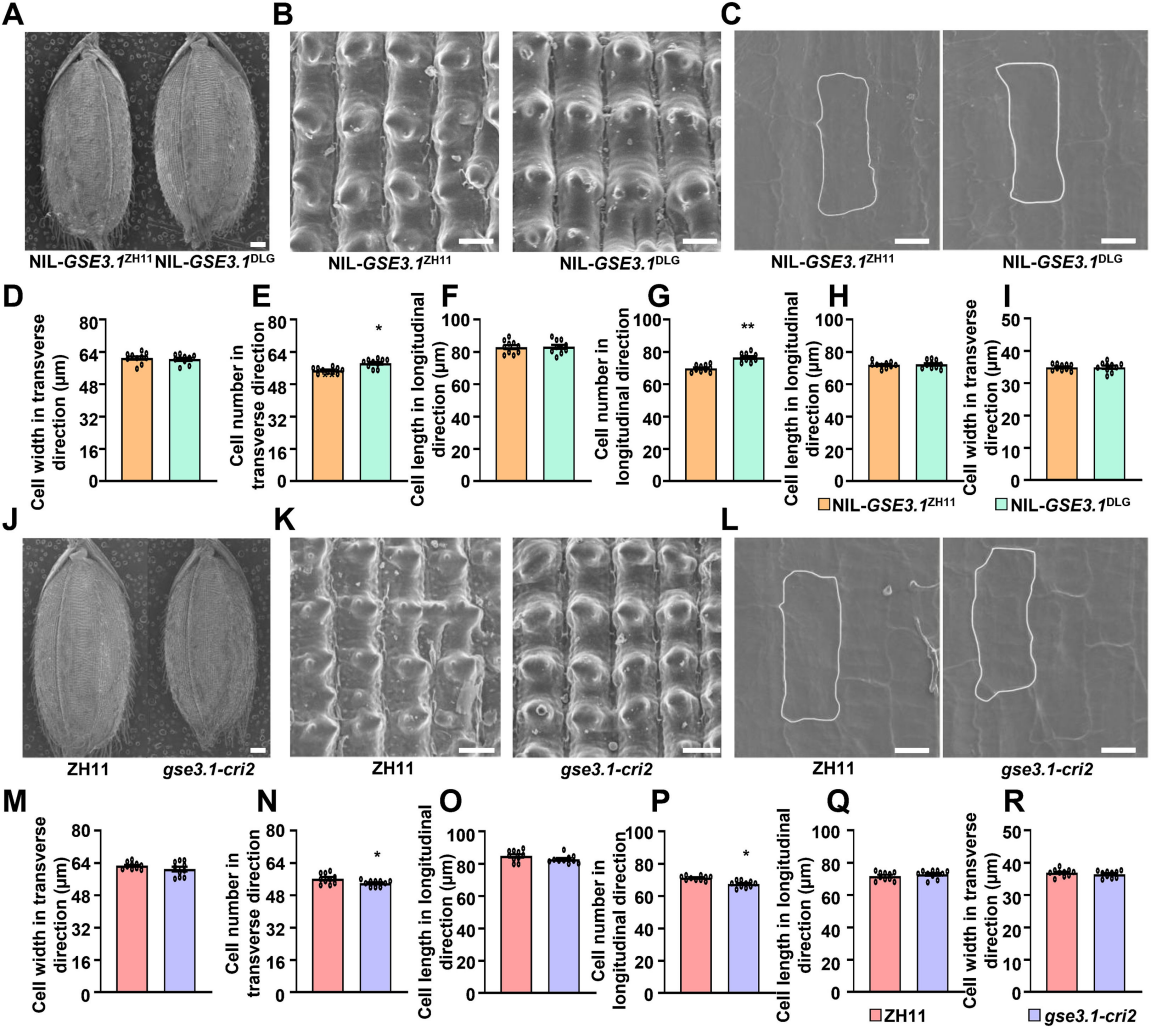
*GSE3.1* regulates grain size by promoting cell division in spikelet hulls. **(A–C)** global outer surface **(A)**, outer surface **(B)** and inner surface **(C)** of NIL-*GSE3.1*^ZH11^ and NIL-*GSE3.1*^DLG^ spikelet hulls. **(D, E)** cell width **(D)** and cell number **(E)** of outer epidermal cells of NIL-*GSE3.1*^ZH11^ and NIL-*GSE3.1*^DLG^ spikelet hulls in transverse direction (n ≥ 10). **(F, G)** cell length **(F)** and cell number **(G)** of outer epidermal cells of NIL-*GSE3.1*^ZH11^ and NIL-*GSE3.1*^DLG^ spikelet hulls in longitudinal direction (n ≥ 10). **(H, I)**, cell length **(H)** and cell width **(I)** of inner epidermal cells of NIL-*GSE3.1*^ZH11^ and NIL-*GSE3.1*^DLG^ spikelet hulls. **(J–L)** global outer surface **(J)**, outer surface **(K)** and inner surface **(L)** of ZH11 and *gse3.1-cri2* spikelet hulls. M-N, cell width **(M)** and cell number **(N)** of outer epidermal cells of ZH11 and *gse3.1-cri2* spikelet hulls in transverse direction (n ≥ 10). **(O, P)** cell length **(O)** and cell number **(P)** of outer epidermal cells of ZH11 and *gse3.1-cri2* spikelet hulls in longitudinal direction (n ≥ 10). **(Q, R)** cell length **(Q)** and cell width **(R)** of inner epidermal cells of ZH11 and *gse3.1-cri2* spikelet hulls. Values **(D-I, M-R)** are given as mean ± SE. **P < 0.01 and *P < 0.05 indicate significant differences between genotypes by Student’s *t*-test. Bars, 500 μm **(A, J)**, 50 μm **(B, K)**, 30μm **(C, L)**.

Rice grain size determination is crucially influenced by the spikelet hull, in which cell division and cell expansion coordinately regulate grain growth (Li et al., 2019). To understand how *GSE3.1* controls grain size, we conducted cell analyses of the NIL-*GSE3.1*^ZH11^ and NIL-*GSE3.1*^DLG^ spikelet hulls. As shown in **Figures 3A, B, D-G**, outer epidermal cell numbers in both longitudinal and transverse directions were significantly increased in the NIL-*GSE3.1*^DLG^ spikelet hulls, while outer epidermal cell length and cell width in the spikelet hulls of NIL-*GSE3.1*^DLG^ and NIL-*GSE3.1*^ZH11^ showed no obvious differences. Additionally, inner epidermal cell length and cell width were almost similar in NIL-*GSE3.1*^DLG^ and NIL-*GSE3.1*^ZH11^ spikelet hulls (**Figures 3C, H, I**). Similarly, cell size of outer and inner epidermal cells in ZH11 and *gse3.1-cri2* spikelet hulls exhibited no obvious changes, while cell numbers of outer epidermal cells in both longitudinal and transverse directions of *gse3.1-cri2* spikelet hulls were significantly decreased compared to those of ZH11 spikelet hulls (**Figures 3J–R**). Therefore, these results revealed that *GSE3.1* regulates grain size predominantly by promoting cell division.

### 2.4 Expression pattern and subcellular localization of *GSE3.1*

**Figure 4:**
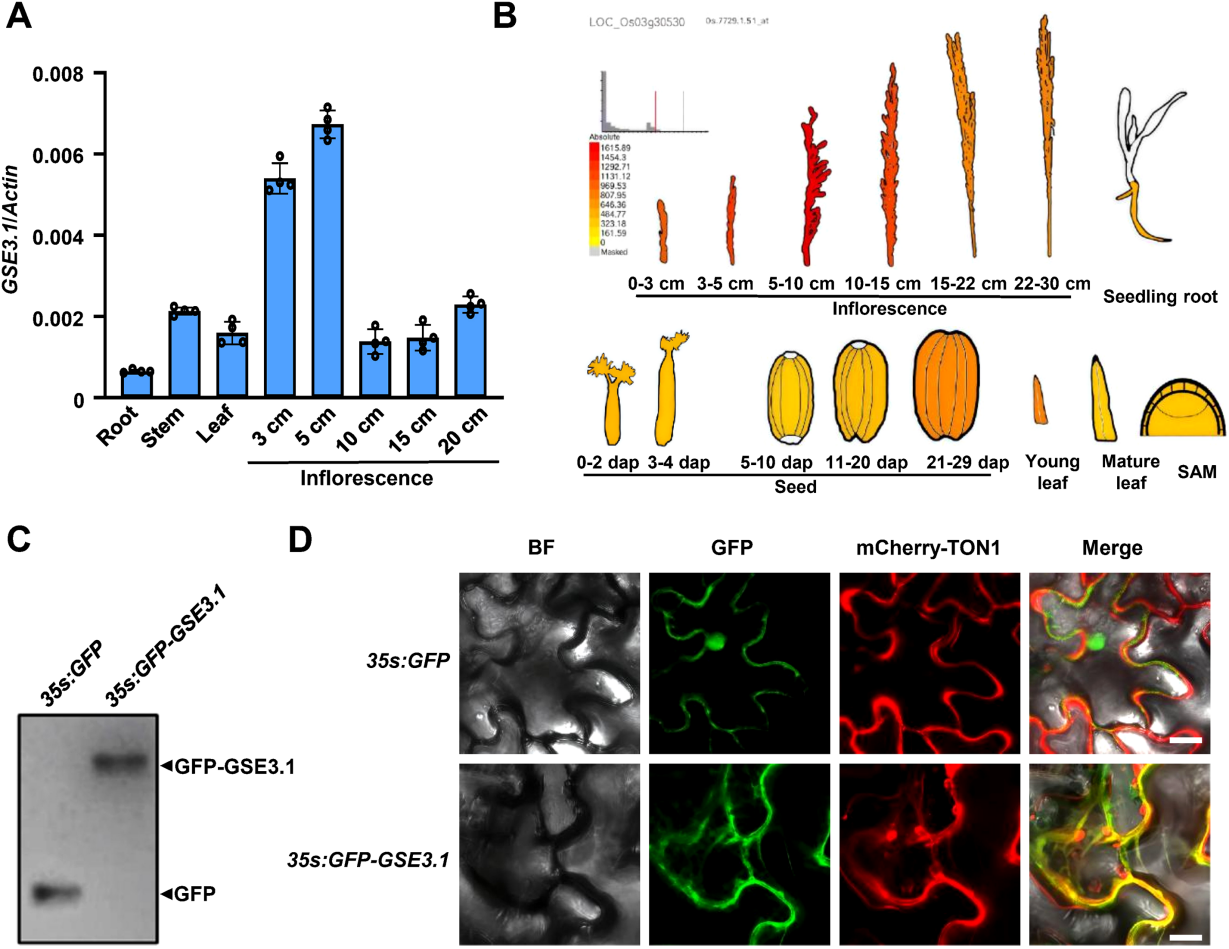
Expression pattern of *GSE3.1* and subcellular localization of GSE3.1. **(A)** expression levels of *GSE3.1* in different rice tissues (n = 4). **(B)** expression patterns of *GSE3.1* in inflorescences, seedling root, developing seed, young leaf, mature leaf and SAM (shoot apical meristem). Data were obtained from botany array resource (http://bar.Utoronto.Ca/efprice/cgi-bin/efpweb.Cgi). **(C)** detection of the GFP-GSE3.1 fusion protein by Western blot analysis. **(D)** subcellular localization *of* GFP-GSE3.1 fusion protein in *N. Benthamiana* leaf epidermal cells. Images show bright field, GFP fluorescence, mCherry fluorescence and the merged view. The GFP-GSE3.1 fusion protein co-localizes with TON1, a microtubule-binding protein. Bar, 20 μm **(D)**.

To elucidate the functions of *GSE3.1* in rice developmental processes, quantitative real-time PCR (qRT-PCR) analysis was performed to determine expression pattern of *GSE3.1*. As shown in **Figure 4A**, *GSE3.1* transcripts were detected in different tissues, including root, stem, leaf and developing inflorescences. Notably, inflorescences at early developing stages (3 cm and 5 cm) had higher expression level of *GSE3.1* than those at later developing stages (10 cm, 15 cm and 20 cm), consistent with the *GSE3.1*/*LOC_Os03g30530* expression profiles from public data resources (**Figure 4B**). Expression pattern of *GSE3.1* further supports the role of *GSE3.1* in grain size control by promoting cell division.

*GSE3.1* encodes a TRM protein with high sequence identity to Arabidopsis TRM1/LNG2 and TRM2/LNG1 proteins (**Supplementary Figure 2**). In Arabidopsis, TRM1 interacts with and targets TON1 to cortical microtubules (Drevensek et al., 2012). To investigate the subcellular localization of GSE3.1 protein, we constructed the *35S:GFP-GSE3.1* and *35S:mCherry*-*TON1* (Arabidopsis *TON1* fused with mCherry) constructs, which were transiently coexpressed in *N. Benthamiana* leaves. The GFP–GSE3.1 fusion protein was detected using a Western blot assay (**Figure 4C**). Green fluorescence was observed and merged with the microtubule-localized protein TON1, indicating GSE3.1 is localized to microtubules (**Figure 4D**).

### 2.5 Application potential of *GSE3.1* for improving grain yield

**Figure 5:**
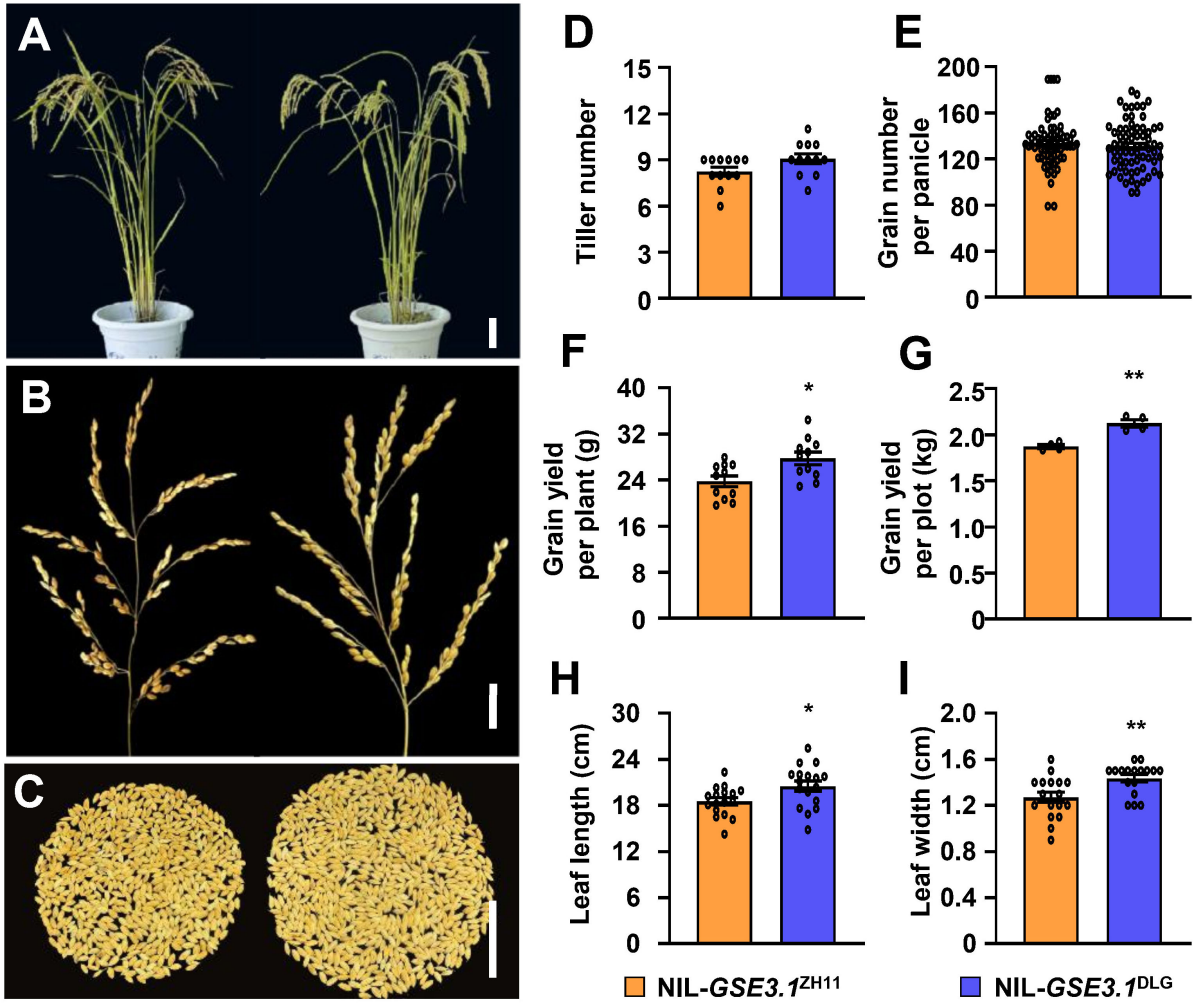
NIL-*GSE3.1*^DLG^ increases grain yield in rice. **(A)** plant morphology of NIL-*GSE3.1*^ZH11^ and NIL-*GSE3.1*^DLG^. **(B)** panicle morphology of NIL-*GSE3.1*^ZH11^ and NIL-*GSE3.1*^DLG^. **(C)** grains per plant of NIL-*GSE3.1*^ZH11^ and NIL-*GSE3.1*^DLG^. **(D)** tiller number of NIL-*GSE3.1*^ZH11^ and NIL-*GSE3.1*^DLG^ (n ≥ 12). **(E)** grain number per panicle of NIL-*GSE3.1*^ZH11^ and NIL-*GSE3.1*^DLG^ (n ≥ 90). **(F)** yield per plant of NIL-*GSE3.1*^ZH11^ and NIL-*GSE3.1*^DLG^ (n ≥ 11). **(G)** plot yield of NIL-*GSE3.1*^ZH11^ and NIL-*GSE3.1*^DLG^ (n = 4). **(H, I)** flag leaf length **(H)** and width **(I)** of NIL-*GSE3.1*^ZH11^ and NIL-*GSE3.1*^DLG^ (n ≥ 17). Values **(D–I)** are given as mean ± SE. **P < 0.01 and *P < 0.05 compared with NIL-*GSE3.1*^ZH11^ by Student’s *t*-test. Bars, 15 cm **(A)**, 3 cm **(B)** and 5 cm **(C)**.

Given that the NIL-*GSE3.1*^DLG^ plants exhibited larger and heavier grains than NIL-*GSE3.1*^ZH11^ plants (**Figures 1G–J**), it is plausible that NIL-*GSE3.1*^DLG^ plants might increase grain yield. No significant differences between NIL-*GSE3.1*^ZH11^ and NIL-*GSE3.1*^DLG^ in terms of overall plant and panicle morphologies, tiller number and grain number per panicle were observed (**Figures 5A, B, D, E**). Grain yield per plant in NIL-*GSE3.1*^DLG^ was increased compared with that in NIL-*GSE3.1*^ZH11^ (**Figures 5C, F**). To investigate the actual grain yield in NIL-*GSE3.1*^DLG^ plants, we performed field plot trials. Overall, NIL-*GSE3.1*^DLG^ plants increased the grain yield by 13.3% in the test plot (**Figure 5G**). In addition, the leaf length and leaf width of NIL-*GSE3.1*^DLG^ were increased compared to those of NIL-*GSE3.1*^ZH11^ (**Figures 5H, I**). It is possible that the large leaves of NIL-*GSE3.1*^DLG^ plants might provide more photosynthetic products for grain growth. Therefore, the *GSE3.1* allele from DLG increases grain size and weight and has the application potential for improving grain yield.

## 3 Discussion

Grain size is one of important agronomic traits that directly determine rice grain yield. In this study, we report that *GSE3.1*, a novel QTL allelic to *GWL1*/*IGL1*, positively regulates grain size by promoting cell division in spikelet hulls. *GSE3.1* encodes a microtubule-localized TRM protein and has a higher expression at early stages of grain development. We also demonstrate that the *GSE3.1*^DLG^ allele, with higher expression of *GSE3.1*, leads to increased grain size and weight, and significantly increases grain yield in the field plot trials compared to the *GSE3.1*^ZH11^ allele. Our findings reveal the molecular mechanism of *GSE3.1* underlying grain size regulation and provide a new allele that has application potential for improving grain yield.

*GWL1*/*IGL1* was previously reported to control grain size in rice. *GWL1* was firstly identified as a homologous gene of *GW7*. A knockout mutant line *wgl1* in the genetic background of Wuyunjing7 (WYJ7)-*DEP1* exhibits short but wide grains, and a *WGL1* overexpression line in WYJ7-*dep1* shows long and narrow grains, similar to related results of *GW7* (Wu et al., 2023). Another study reported that *IGL1* functions as a positive regulator for grain length. Knockout mutants and overexpression lines of *IGL1* in the Nippobare (NIP) background result in short and long grains, respectively (Niu et al., 2024). Given the two published studies, *GWL1*/*IGL1* is a positive regulator of grain length, consistent with our results of *GSE3.1*. The latter study didn’t present grain width phenotypes with regard to the *IGL1* knockout mutants and overexpression lines, while the former regarded *GWL1* as a negative regulator for grain width, which contradicts with our results. This seemingly contradictory phenomenon is not rare in rice because some factors, especially genetic background, may lead to contradictory phenomena in grain size control. Overexpression of *GSE9* in ZH11 and NIP results in longer and wider grains, while introgression of *GSE9* into Kasalath leads to shorter and wider grains (Chen et al., 2023). The *GL3.1*/*OsPPKL1* knockout mutant in Dongjin background produces longer grains (Zhang et al., 2012), whereas a recent study revealed that its knockout mutant in the ultra-large grain accession (ULG) background results in shorter grains (Li et al., 2024). Therefore, the diverse function of *GWL1*/*GSE3.1* in grain width control might result from different genetic backgrounds. Future molecular and genetic analyses combined with CRISPR/Cas9 knockout system are necessary to solve this problem.

Variations of cis-regulatory elements in promoter regions can influence binding of core transcription factors, which significantly influences gene expression (Wittkopp and Kalay, 2011). By comparing sequence variations of *GSE3.1* promoters, *GSE3.1* expression and *GSE3.1* promoter activity in the NILs, we reveal that the *GSE3.1* promoter from DLG causes increased *GSE3.1* expression, which is responsible for the large and heavy grains in the NIL-*GSE3.1*^DLG^. Whether the variations of DLG promoter affects the binding of possible transcription factors needs further investigation. In addition, DLG has an A/G transition in the third exon (A1176G), leading to an Asn/Ser amino acid substitution (Asn338Ser). GSE3.1/WGL1 has been reported to interact with OsTON1b and OsTON2, and future researches are needed to test if this amino acid substitution could impact interactions of GSE3.1 with OsTON1b and OsTON2. Therefore, it would be worth studying the variations in *GSE3.1* promoter and possible bound transcription factors, as well as the amino acid substitution (Asn338Ser) and its possible functional differences in the future, which will enrich the molecular mechanisms of *GSE3.1* in grain size control.

GSE3.1 protein homologs are found in different plant species (**Supplementary Figure 2**). Several studies have shown that TRM proteins participate in seed/grain size regulation. Arabidopsis TRM1/LNG2 and TRM2/LNG1, rice GL7/GW7/SLG7 and maize ZmLNG1 positively regulate seed/grain length but negatively regulate seed/grain width. However, our study demonstrates that GSE3.1 acts as a positive regulator in both grain length and grain width. It seems that the former plant TRM proteins, especially rice GW7/GL7/SLG7, contradict GSE3.1 with respect to seed/grain width. Generally, protein homologs, such as GW5 and GW5L, two calmodulin homologs, have similar functions in controlling rice grain size (Duan et al., 2017; Liu et al., 2017; Tian et al., 2019). However, several studies have shown that protein homologs also have diverse roles in regulating grain size. The Kelch-like repeat domain-containing phosphatase OsPPKL2 is a positive regulator of grain length, while OsPPKL1 and OsPPKL3, the closest homologs of OsPPKL2, act as negative regulators of grain length (Zhang et al., 2012). Similarly, the SQUAMOSA Promoter-Binding-Like (SPL) transcription factors, OsSPL13 and OsSPL16, have opposite effects on grain length determination (Si et al., 2016; Wang et al., 2012). In our study, NIL-*GSE3.1*^DLG^, which has higher *GSE3.1* expression, shows increased grain length and grain width compared to NIL-*GSE3.1*^ZH11^. In addition, grain size analyses in genomic complementation test and *GSE3.1* knockout mutants further prove that GSE3.1 is a positive regulator in both grain length and grain width. These results suggest that GSE3.1 has unique advantages over GL7/GW7/SLG7 in improving grain weight and yield in rice.

On cellular level, rice grain size is crucially influenced by cell division and cell expansion in spikelet hulls (Li et al., 2019). TRM proteins in several species have been shown to regulate organ size, especially seed/grain size. In rice, three independent studies with regard to the identical gene *GW7*/*GL7*/*SLG7* were almost simultaneously reported. However, there is a controversy about cellular mechanisms of *GW7*/*GL7*/*SLG7* in controlling grain size. The slender grains of gain-of-function *GL7*/*SLG7* result from increased cell length and decreased cell width (Wang et al., 2015; Zhou et al., 2015), demonstrating that *GL7*/*SLG7* regulates grain size by affecting cell expansion rather than cell division. Another study revealed that upregulation of *GW7* correlates with slender grains due to increased cell division in the longitudinal direction and decreased cell division in the transverse direction (Wang et al., 2015), suggesting that *GW7* regulates grain size by affecting cell division rather than cell expansion. Further cellular analyses of *GL7*/*GW7*/*SLG7* are needed to resolve this controversy. In dicots, Arabidopsis *LNG1*/*TRM2* positively regulates cell expansion rather than cell division along the proximal-distal axis to control leaf shape (Lee et al., 2006), while tomato *SlTRM5* regulates cell division in the proximal-distal and medial-lateral directions to control fruit shape (Wu et al., 2018). Given the above studies, it can be concluded that TRM proteins regulate organ size depending on both cell division and cell expansion. It is well-known that TRM proteins associate with microtubules (Drevensek et al., 2012; Lazzaro et al., 2018), which constitute the structural framework of plant cells and play pivotal regulatory roles in numerous cellular processes, including cell division and cell expansion (Ehrhardt and Shaw, 2006). Thus, it is reasonable that TRM proteins regulate organ size by affecting cell division and cell expansion. In our study, cellular analyses of both NILs and knockout mutant revealed that *GSE3.1* positively regulates grain size by promoting cell division in spikelet hulls. In addition, higher expression of *GSE3.1* was detected at early stages of grain development, further supporting the role of *GSE3.1* in cell division. Therefore, our study is a good example of TRM proteins controlling organ size by cell division, and it will be worth investigating cellular mechanisms of more TRM or cytoskeleton-related proteins in control of organ size.

Grain size usually negatively correlates with grain number, which is also referred to as the trade-off effect (Sadras, 2007). For example, overexpression of the histone acetyltransferase gene *GSE3* exhibits increased grain size but decreased grain number, thus resulting in unchanged grain yield (Huang et al., 2024). Strikingly, the NIL-*GSE3.1*^DLG^ plant produces large and heavy grains without decreasing tiller number and grain number per panicle, and finally increased grain yield by 13.3% in the plot trials, which suggests the application potential of *GSE3.1* in breeding high-yield rice varieties.

In summary, our findings report the identification and characterization of a novel QTL *GSE3.1* in grain size control. *GSE3.1* encodes a TRM protein with subcellular localization to microtubules and positively regulates grain size by promoting cell division. Furthermore, the *GSE3.1* allele from DLG significantly increases grain yield in field plot trials. Therefore, our findings reveal the molecular mechanism of grain size control in rice and provide a new allele that could be utilized for breeding high-yield varieties in rice.

## 4 Materials and methods

### 4.1 Plant materials and growth conditions

The japonica varieties ZH11 and DLG were used as recipient parent and donor, respectively. ZH11 and DLG were crossed to produce the F_1_ population, which was subsequently backcrossed with ZH11 to generate the BC_3_F_3_ and BC_4_F_3_ populations. The BC_3_F_3_ populations were used for fine mapping. NIL-*GSE3.1*^ZH11^ and NIL-*GSE3.1*^DLG^ were obtained in BC_4_F_3_ populations. The *gse3.1-cri1* and *gse3.1-cri2* mutants were generated using CRISPR/Cas9 technology. Rice plants were cultivated in open fields at Lingshui and Beijing during the natural growing seasons. *N. benthamiana* plants were grown in a greenhouse under a 16-h light/8-h dark photoperiod.

### 4.2 Morphological analysis

After plants and grains reached maturity, they were photographed and measured. Grains from the main panicles were scanned using a scanner (MICROTEK ScanMarker i560). Grain length and width were then measured using the Rice Test System (WSEEN). To determine grain weight, one hundred dry grains were weighed, and the average value was calculated from three biological replicates.

### 4.3 Cellular analysis

SEM and Image J software were used for cellular analyses of mature grains according to a previous study (Li et al., 2025). For outer epidermal cell analysis, intact grains were observed with SEM. We measured grain width and counted total cell number in transverse direction and obtained cell width by dividing the former by the latter. We also measured grain length and counted total cell number in longitudinal direction and obtained cell length by dividing the former by the latter. For inner epidermal cell analysis, the lemmas were separated from grains, then divided and subjected to observation with SEM. We measured lemma width and counted total cell number in transverse direction and obtained cell width by dividing the former by the latter. We also measured lemma length and counted total cell number in longitudinal direction and obtained cell length by dividing the former by the latter.

### 4.4 Vector construction and transformation

Plasmids were constructed using the Uniclone One−Step Seamless Cloning Kit (Genesand, SC612). The genomic sequence of *GSE3.1* was amplified with primers g*GSE3.1*-F and *gGSE3.1*-R and inserted into the *pMDC99* vector to generate the g*GSE3.1* plasmid. The coding sequence (CDS) of *GSE3.1* was amplified with primers GFP-*GSE3.1*-F and GFP-*GSE3.1*-R and inserted into the *pMDC43* vector to generate the *35S:GFP-GSE3.1* plasmid. The GSE3.1 knockout plasmid was constructed using primers *gse3.1*-cri-F/F0 and *gse3.1*-cri-R/R0. All constructs were transformed into the japonica variety ZH11 via *Agrobacterium tumefaciens* strain GV3101 to obtain transgenic plants. Primers used for PCR amplification are listed in **Supplementary Table 3**.

### 4.5 Analysis of *GSE3.1* promoter activity

The promoter sequences of *GSE3.1* from DLG and ZH11 were cloned into the *pGreenII0800:LUC* vector to generate *proGSE3.1*^ZH11^*:LUC* and *proGSE3.1*^DLG:^*LUC*, respectively, which were transformed into *Agrobacterium* GV3101 competent cells. Positive colonies were cultured at 28 °C with shaking at 250 rpm for 24 ;h. Bacterial cells were collected, resuspended in 10 mM MgCl_2_ containing 2 μL mL&^-^¹ of 0.1 M acetosyringone (AS), and incubated for 3 h. Then, 200 ;μL of the suspension was infiltrated into 4-week-old *N. benthamiana* leaves. After 2 ;d, luciferase activity was quantified to evaluate promoter-driven expression levels.

### 4.6 RNA extraction and quantitative real-time PCR

Total RNA was extracted from different rice tissues using the RNAprep Pure Kit (TIANGEN, DP439). First strand cDNA was synthesized with the cDNA Synthesis Kit (Vazyme, R211). Quantitative real-time PCR was performed on a LightCycler 480 system (Roche, Switzerland) using SYBR qPCR Mix (Genstar, A301-10). Rice *Actin1* was used as the internal control. Primer sequences are provided in **Supplementary Table 3**.

### 4.7 Subcellular localization of *GSE3.1*

The full-length CDS of Arabidopsis *TON1* was cloned into the *pCAMBIA1300-mCherry* vector to generate *35S:mCherry-TON1*. The full-length CDS of *GSE3.1* was cloned into the *pMDC43* vector to generate *35S:GFP-GSE3.1*. The construct pairs were transformed into *Agrobacterium* GV3101 competent cells. Positive single colonies were cultured overnight, and bacterial suspensions were adjusted to OD600 = 0.6. Leaves of 4-week-old *N. benthamiana* plants were infiltrated, and subcellular localization was observed after 2 d using a laser-scanning confocal microscope (Zeiss LSM710).

### 4.8 Statistical analysis

Unless otherwise indicated, data were presented as mean ± SE. Statistical analyses were performed using GraphPad Prism 8 (GraphPad Software, Inc., San Diego, CA, USA). Details of statistical tests were provided in the figure legends. P-values were calculated using two−tailed unpaired Student’s t-tests. The sample size was not predetermined by any statistical method.

## Data Availability

Publicly available datasets were analyzed in this study. This data can be found here: https://rice.uga.edu/cgi-bin/ORF_infopage.cgi?orf=LOC_Os03g30530.
