# Supplementary material for: A novel QTL GSE3.1 regulates grain size and weight in rice
Source: Front Plant Sci. 2026 Mar 11;17:1784176. doi: 10.3389/fpls.2026.1784176 (PMC13013363; doi:10.3389/fpls.2026.1784176)
Supplement: Supplementary file 1 [file DataSheet1.pdf]

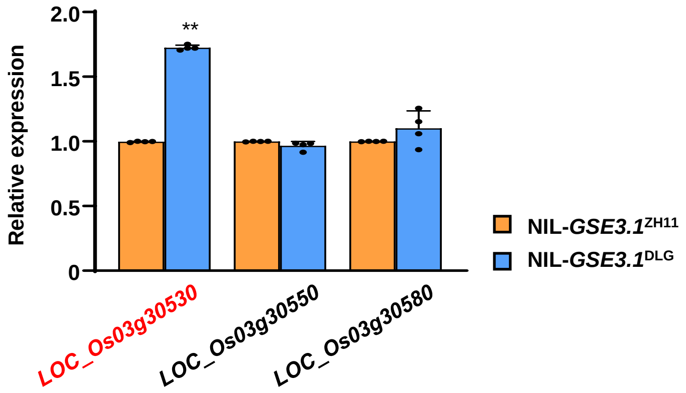

Supplementary Figure 1 | Expression levels of genes in NIL-*GSE3.1*ZH11 and NIL-*GSE3.1*DLG. The three genes havouring genomic variations in the promoter, CDS and 3'UTR regions between ZH11 and DLG were chosen for qPCR analysis. Only *LOC_Os03g30530*, highlighted in red, exhibited significantly differential expression between NIL-*GSE3.1*ZH11 and NIL-*GSE3.1*DLG. **P < 0.01 indicates significant differences compared with NIL-*GSE3.1*ZH11 by student's *t*-test.

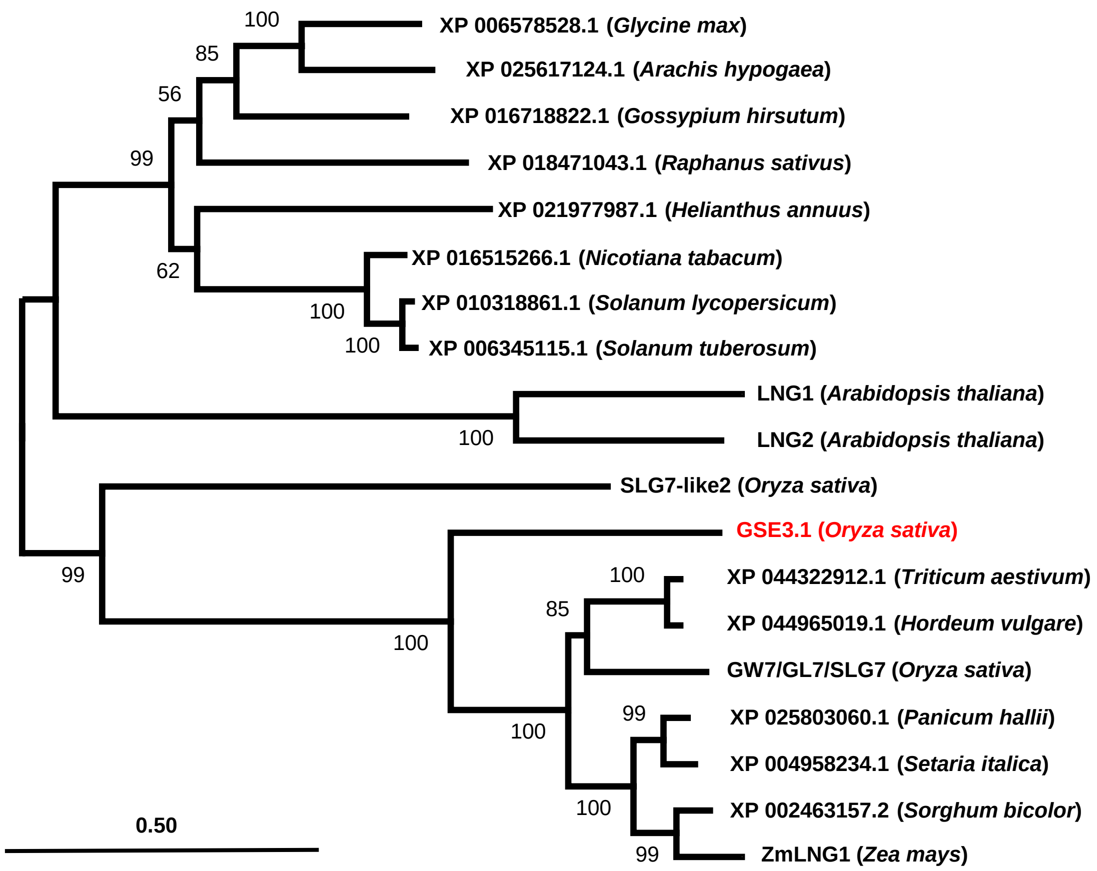

Supplementary Figure 2 | The phylogenetic tree of GSE3.1. The tree was constructed by the Neighbor-Joining method in MEGA12.0 program. The full-length sequences of GSE3.1 homologs in various species were used to construct the phylogenetic tree. The evolutionary distances were computed using the JTT matrix-based method with a gamma distribution. Numbers at nodes indicate percentage of 6,000 bootstrap replicates.

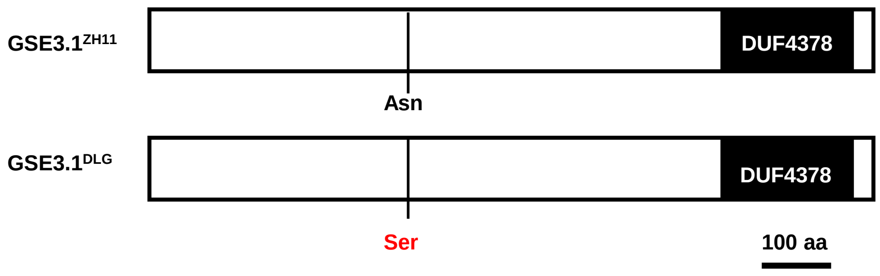

Supplementary Figure 3 | The protein diagrams of GSE3.1^{ZH11} and GSE3.1^{DLG}. The A/G transition in the third exon (A1176G) of DLG leads to a Asn/Ser amino acid substitution (Asn338Ser), which are shown in red. Bar, 100 aa.

Supplementary Table 1. Gene list in the 153.8 kb genomic region.

**Gene ID**	**Description**
LOC_Os03g30460	vacuolar protein sorting-associated protein 52, putative, expressed
LOC_Os03g30470	CHIT4 - Chitinase family protein precursor, expressed
LOC_Os03g30500	hypothetical protein
LOC_Os03g30510	expressed protein
LOC_Os03g30519	Awn3-1
LOC_Os03g30530	GWL1/IGL1, SLG7-like protein, Regulation of grain shape
LOC_Os03g30550	RNA-binding protein-like, putative, expressed
LOC_Os03g30570	OsNINJA3
LOC_Os03g30580	expressed protein
LOC_Os03g30590	expressed protein
LOC_Os03g30600	expressed protein
LOC_Os03g30610	ubiquinone oxidoreductase, putative, expressed
LOC_Os03g30620	expressed protein
LOC_Os03g30630	expressed protein
LOC_Os03g30640	expressed protein
LOC_Os03g30650	expressed protein
LOC_Os03g30670	expressed protein
LOC_Os03g30680	expressed protein
LOC_Os03g30690	expressed protein
LOC_Os03g30700	retrotransposon protein, putative, unclassified, expressed
LOC_Os03g30710	retrotransposon protein, putative, unclassified, expressed
LOC_Os03g30720	retrotransposon protein, putative, unclassified, expressed
LOC_Os03g30730	retrotransposon protein, putative, unclassified, expressed
LOC_Os03g30740	expressed protein
LOC_Os03g30744	expressed protein
LOC_Os03g30750	hypothetical protein

The descriptions of genes were obtained from China Rice Data Center (https://www.ricedata.cn/gene/).

Supplementary Table 2. genomic variations of ZH11 and DLG in the 153.8 kb genomic region.

**Variation**	**Gene ID**	**Position**
17420383 A-AG	*LOC_Os03g30530*	promoter
17421125 A-C	*LOC_Os03g30530*	promoter
17421863 A-G	*LOC_Os03g30530*	promoter
17421889 A-ATTCC	*LOC_Os03g30530*	promoter
17424236 A-G	*LOC_Os03g30530*	CDS
17437173 C-T	*LOC_Os03g30550*	3'UTR
17454933 A-C	*LOC_Os03g30580*	3'UTR

The variations in the promoter, CDS, exon-intron boundary, 5' UTR and 3' UTR regions of genes were shown. The variation column includes location on Chr.3 of the variation, and its corresponding nucleotides of ZH11 and DLG.

Supplementary Table 3 | Primers used in this study

**Primer Name**	**Primer sequence**
**Primers for mapping**	
M3-1-F	CTTTGGCTCTGTAAACCAC
M3-1-R	GAATATAACCTCAGCATCCG
M3-2-F	ATGTCGCATCGGATATACC
M3-2-R	CGAGACAAATCTTTTAAGTGTA
M3-3-F	TTGTTCAATAGGCATACAAGC
M3-3-R	TTATAGGAAAAGCAACAGAGCA
M3-4-F	TATTCCACAAATGCAGGCCAA
M3-4-R	CAACTGAATAACTCCTGGGTG
M3-5-F	AAATATAGTCATTAATCGGCAT
M3-5-R	CTCTAGCATGTTATCGTGT
M3-6-F	CTAGGACATAGACTGCCCTCA
M3-6-R	GAATGTGACATCCCGGCTC
M3-7-F	ATGCTTTGGAATTATTGCCAT
M3-7-R	TTATGTTGACTTGGGCATGTT
M3-8-F	CATCACTGCAAATGACACC
M3-8-R	CTTCAGCCAAACAGGTCTT
M3-9-F	ACAAGTATAAAATTTCAACAA
M3-9-R	ATTGATGTTTTATGCAGCTA
M3-10-F	AGGCTTAAAAGATTCTTCTCG
M3-10-R	TCTCTCCCCTATTCCTTCCAA
**Primers for *gGSE3.1* construct**	
gGSE3.1-F	CATGATTACGAATTCGAGCTCTGTGTTAACTACTTCCCGAGA
gGSE3.1-R	TCTAGAGGATCCCCGGGTACCTTCAGTTGCCCTGTTCGTC
**Primers for *GFP-GSE3.1* construct**	
GFP-GSE3.1-F	GATGAACTATACAAAGGCGCGCCAATGCCGTCCCGGATGATGCA
GFP-GSE3.1-R	CGATCGGGGAAATTCGAGCTCCTATATGCTGATGAAAGACA
**Primers for *proGSE3.1:LUC* construct**	
proGSE3.1-F	GGCGAATTGGGTACCACACTGGTCCCTGGAGTACA
proGSE3.1-R	AGAACTAGTGGATCCCCATGGCAGAACCAGGCTAA
**Primers for *gse3.1-cri* construct**	

gse3.1-*cri*-F	AATAATGGTCTCAGGCGACAAAGAAATCCAAAGAAA
gse3.1-*cri*-F0	GACAAAGAAATCCAAAGAAAGTTTTAGAGCTAGAAATAGC
gse3.1-*cri*-R	AAAGAAATCCGGTACAAAACGCTTCTTGGTGCC
gse3.1-*cri*-R0	ATTATTGGTCTCTAAACAAAGAAATCCGGTACAAAA
**Primers for qRT-PCR**	
GSE3.1-qRT-F	AGAGCAACTCTGACAACGATAA
GSE3.1-qRT-R	TCTTGTCTGCAGGTAGAATTGT
LOC_Os03g30550-qRT-F	CACTCTTCCTGAAACTGATCCT
LOC_Os03g30550-qRT-R	GCTCCTCTTTCGTCATTTGTAC
LOC_Os03g30580-qRT-F	TGTTCCATCTGGTTGAGCTAAT
LOC_Os03g30580-qRT-R	CGAACTACATACAAGTAGGGCT
Actin-qRT-F	TGCTATGTACGTCGCCATCCAG
Actin-qRT-R	AATGAGTAACCACGCTCCGTCA
**Primers for *TON1-mCherry* construct**	
TON1-*mCherry*-F	CAAATCGACTCTAGAAAGCTTATGTCGGCGAAGCTTTTGTATA
TON1-*mCherry*-R	CCTTGCTCACCATGGTACCGCAATGAAAAAGCTGCCTTGGT
